# Copper Deficiency, Lead, and Paraoxonase: Li et al. Respond

**DOI:** 10.1289/ehp.10151R

**Published:** 2007-07

**Authors:** Wan-Fen Li, Hung-Yi Chuang

**Affiliations:** Division of Environmental Health and Occupational Medicine, National Health Research Institutes, Zhunan, Taiwan; Graduate Institute of Public Health, Kaohsiung Medical University, Kaohsiung, Taiwan, E-mail: ericch@cc.kmu.edu.tw

We appreciate the opportunity to discuss the issue raised by Klevay in his letter. Klevay suggested that the decrease of PON1 activity in lead workers, as we reported in our article (Li et al. 2006), might be due to copper deficiency induced by lead intoxication. Because the scope of our study did not include the interaction between lead and copper, we did not measure plasma copper level, ceruloplasmin, or superoxide dismutase (SOD) activity in our cohort. Therefore, it is not possible to test Klevay’s hypothesis at this point.

Although animal studies have shown that lead ingestion caused a decrease in blood copper level in cattle (Doyle and Younger 1984) and weanling rats (Mylroie et al. 1986), the relationship between lead exposure and copper deficiency in humans is less evident. Although Ito et al. (1985) and Patil et al. (2006) showed a decrease of SOD activity in lead workers, results of other studies did not agree with such findings (el-Gazzar and Hamid 1998; Oktem et al. 2004). Whether long-term lead exposure induces copper deficiency in humans is still in question.

To our knowledge, there are very few studies dealing with the effects of copper on PON1 activity. Debord et al. (2003) showed that copper ion had weak inhibitory effects on PON1 activity *in vitro*, whereas Klevay (2004) claimed that a copper deficiency diet caused PON1 activity reduction in rats. With little information available, it is difficult to conclude whether copper has any effects on PON1 activity in humans.

However, because of the link between copper deficiency and cardiovascular disease, we agree with Klevay that in the future, the association between lead exposure, copper deficiency, and serum PON1 activity should be examined.
